# Genome-Wide Identification of a Regulatory Mutation in BMP15 Controlling Prolificacy in Sheep

**DOI:** 10.3389/fgene.2020.00585

**Published:** 2020-06-19

**Authors:** Louise Chantepie, Loys Bodin, Julien Sarry, Florent Woloszyn, Florence Plisson-Petit, Julien Ruesche, Laurence Drouilhet, Stéphane Fabre

**Affiliations:** GenPhySE, Université de Toulouse, INRAE, ENVT, Castanet-Tolosan, France

**Keywords:** GWAS, major gene, prolificacy, sheep, BMP15

## Abstract

The search for the genetic determinism of prolificacy variability in sheep has evidenced several major mutations in genes playing a crucial role in the control of ovulation rate. In the Noire du Velay (NV) sheep population, a recent genetic study has evidenced the segregation of such a mutation named *FecL*^*L*^. However, based on litter size (LS) records of *FecL*^*L*^ non-carrier ewes, the segregation of a second prolificacy major mutation was suspected in this population. In order to identify this mutation, we have combined a case/control genome-wide association study with ovine 50k SNP chip genotyping, whole genome sequencing, and functional analyses. A new single nucleotide polymorphism (OARX:50977717T > A, NC_019484) located on the X chromosome upstream of the *BMP15* gene was evidenced to be highly associated with the prolificacy variability (*P* = 1.93E-11). The variant allele was called *FecX*^*N*^ and shown to segregate also in the Blanche du Massif Central (BMC) sheep population. In both NV and BMC, the *FecX*^*N*^ allele frequency was estimated close to 0.10, and its effect on LS was estimated at +0.20 lamb per lambing at the heterozygous state. Homozygous *FecX*^*N*^ carrier ewes were fertile with increased prolificacy in contrast to numerous mutations affecting *BMP15*. At the molecular level, *FecX*^*N*^ was shown to decrease *BMP15* promoter activity and supposed to impact *BMP15* expression in the oocyte. This regulatory action was proposed as the causal mechanism for the *FecX*^*N*^ mutation to control ovulation rate and prolificacy in sheep.

## Introduction

There is now an accumulation of evidence that the oocyte plays a central role in controlling the ovarian folliculogenesis from the early stages up to ovulation. Among the local factors produced by the oocyte itself, members of the bone morphogenetic protein/growth and differentiation factor (BMP/GDF) family play an integral role in this control (Persani et al., 2014). Among them, the most important are surely *BMP15* and *GDF9*. Knockout mice models gave the first evidence of the importance of these two oocyte-derived factors acting individually as homodimers and/or through a synergistic cooperation to control the ovarian function (Elvin et al., 1999; Yan et al., 2001).

In humans also, a focus was done on *BMP15* and *GDF9* about their implication in various ovarian dysfunctions. Indeed, numerous heterozygous missense mutations have been identified in both genes associated with primary or secondary amenorrhea in different cohorts of women affected by primary ovarian insufficiency (POI) all over the world. Particularly, the 10-fold higher prevalence of *BMP15* variants among patients with POI compared with the control population supports the causative role of these mutations (Persani et al., 2014). Alterations of *BMP15* and *GDF9* were also searched in association with polycystic ovary syndrome (PCOS). Here, again, several missense variants were discovered in both genes, but the pathogenic role of these mutations remains controversial in the etiology of this syndrome. However, several studies have reported an aberrant expression of *BMP15* and *GDF9* in the ovary of PCOS patients (Teixeira Filho et al., 2002; Wei et al., 2014). Interestingly, some *BMP15* polymorphisms situated in the 5′UTR are significantly associated with the over-response to recombinant FSH applied during assisted reproductive treatment and with the risk to develop ovarian hyper-stimulation syndrome (OHSS, Morón et al., 2006; Hanevik et al., 2011). Finally, polymorphisms in *BMP15* and *GDF9* genes were also searched in association with dizygotic twinning in humans. If no convincing results were obtained for *BMP15*, some loss-of-function variants of *GDF9* were observed significantly more frequently in mothers of twins compared to the control population (Palmer et al., 2006).

In parallel, the search for the genetic determinism of ovulation rate and prolificacy variability in sheep has also highlighted the crucial role of *BMP15* and *GDF9* by evidencing numerous independent loss-of-function mutations, all altering the coding sequence of these two genes (Persani et al., 2014; Abdoli et al., 2016). Depending on the mutation and its hetero- or homozygous state, the phenotype controlled by these mutations in *BMP15* and *GDF9* goes from the early blockade of folliculogenesis and subsequent sterility to an extraordinary increase of ovulation rate (OR) and, thus, litter size (LS) of carrier ewes (Galloway et al., 2000; Hanrahan et al., 2004; Silva et al., 2011; Demars et al., 2013). Thus, sheep exhibiting an extremely high prolificacy are of great interest for identifying genes and mutations involved in molecular pathways controlling ovarian function. These animal models have a double interest in agriculture for genetic improvement of the prolificacy and in human clinics for providing valuable candidate genes in the genetic determinism of female infertility or subfertility as described above.

The Noire du Velay (NV) population is a French local sheep breed mainly reared in the Haute-Loire and Loire departments. Ewes present naturally out-of-season breeding ability, very good maternal characteristics, and a quite high prolificacy (mean LS = 1.62 lamb per lambing). Large variation in LS has been observed in this breed, and a recent genetic study has evidenced the segregation of an autosomal mutation named *FecL*^*L*^ controlling this trait (Chantepie et al., 2018). This variant, located in intron 7 of the *B4GALNT2* gene and associated with its ectopic ovarian expression, was originally discovered in the Lacaune meat sheep breed, increasing OR and prolificacy (Drouilhet et al., 2013). For the segregation study, more than 2700 NV ewes with LS records were genotyped at the *FecL* locus (Chantepie et al., 2018). Surprisingly, the distribution of LS and the existence of highly prolific ewes among the *FecL*^*L*^ non-carriers have suggested the possible segregation of a second prolificacy major mutation in this population as previously observed in the Lacaune breed carrying both *FecL*^*L*^ and *FecX*^*L*^ (Bodin et al., 2007; Drouilhet et al., 2013). In order to validate this hypothesis, after specific genotyping excluding all other known mutations affecting OR and LS and segregating in French sheep populations, we have performed a genome-wide association study (GWAS) based on a case/control design. Completed by the whole-genome sequencing of two specifically chosen animals based on their genotypes, we have identified a new regulatory variant called *FecX*^*N*^ proposed to affect the oocyte-dependent expression of *BMP15* in association with increased prolificacy in sheep.

## Materials and Methods

### Animals

Ewes (*Ovis aries*) from the NV breed (*n* = 2266) were genotyped on blood DNA at the *FecL* locus as previously described (Chantepie et al., 2018). In order to test the hypothesis of the segregation of a second major mutation controlling LS in this breed, a first set of 80 ewes with at least 5 LS records (mean LS = 1.84, ranging from 1.00 to 3.50) were selected among the *FecL*^+^ homozygous genotype (*n* = 2151, mean LS = 1.58). Subsequently, for the NV breed, the effect of the *FecX*^*N*^ mutation on LS was estimated on 2252 ewes, considering the genotype at the *FecL* locus. The presence of the *FecX*^*N*^ mutation in other breeds was checked on a diversity panel of 725 animals from 26 French sheep breeds (Rochus et al., 2018; Table 3). For the BMC population, the effect of the *FecX*^*N*^ mutation on LS was estimated on 2456 ewes. For gene expression analysis, 10 homozygous ewes at the *FecX*^*N*^ locus (five carriers and five non-carriers of the *N* allele) were bought from private breeders (six NV and four BMC) and reared at the INRA experimental facility (agreement number: D3142901). All experimental procedures were approved (approval number 01171.02) by the French Ministry of Teaching and Scientific Research and local ethical committee C2EA-115 (Science and Animal Health) in accordance with the European Union Directive 2010/63/EU on the protection of animals used for scientific purposes.

### Biological Samples

All blood sampling from the numerous sheep breeds studied were collected from the jugular vein (5 ml per animal) by the Venoject system with EDTA and directly stored at −20°C for further use. Part of these blood samples (GWAS and diversity panel) was used for extraction of genomic DNA as described (Bodin et al., 2007). All other samples were used for direct genotyping on whole blood without DNA purification (Chantepie et al., 2018).

For ovary collection and oocyte isolation, the estrus cycles of all adult NV and BMC ewes were synchronized with intravaginal sponges impregnated with flugestone acetate (FGA, 30 mg, CEVA) for 14 days. Ovaries were collected at slaughtering during the follicular phase 36 h after FGA sponge removal. Cumulus-oocyte complexes (COC) were immediately recovered from all visible 1–3 mm follicles by aspiration using a 1 ml syringe with a 26 G needle and placed in McCoy’s 5A culture medium (Sigma-Aldrich). COC were mechanically dissociated by several pipetting and washing cycles in 150 μl drops of McCoy’s 5A medium, and finally, denuded oocytes devoid of granulosa cells were recovered in 1X PBS. Only intact oocytes with a good homogeneity of the cytoplasm were grouped to obtain two to three pools of five oocytes per animal and stored at −80°C before RNA extraction.

### Genotyping Analyses

The *FecL*^*L*^ mutation (OAR11:36938224T > A, NC_019468) was genotyped directly on whole blood samples by the KAPA-KASP assay as previously described (Chantepie et al., 2018). As a prerequisite before GWAS, a set of 30 highly prolific *FecL^+^/FecL^+^* ewes was controlled for the absence of other evidenced major mutations affecting sheep prolificacy in French populations. Using the same KAPA-KASP assay, *FecX*^*L*^ and *FecX*^*Gr*^ alleles in *BMP15* were genotyped as described (Chantepie et al., 2018). *FecB*^*B*^ in the exon 7 of the *BMPR1B* gene (OAR6:29382188A > G, NC_019463.1) was genotyped using forced restriction fragment length polymorphism (RFLP) as described by Wilson et al. (2001).

Whole-genome genotyping was performed on 80 ovine genomic DNA samples using the OvineSNP50 Genotyping Beadchip from Illumina according to the manufacturer’s protocol at the Laboratoire d’Analyses Geìneìtiques pour les espèces Animales (LABOGENA, Jouy en Josas, France)^1^. From the data set, individuals with a call rate < 0.98 were excluded. SNP exclusion thresholds were call frequency < 0.95 and minor allele frequency (MAF) < 0.01 or a significant deviation from Hardy–Weinberg equilibrium (HWE) in the controls (*p* < 1.10^–6^). Non-polymorphic SNP positions and markers with no position on the OARv3.1 reference genome map were also discarded. Finally, from the available design of 54,241 SNPs available on the Illumina OvineSNP50 Beadchip and 80 selected NV ewes, the final data set was reduced to 47,446 SNPs analyzed in 79 individuals.

The *FecX*^*N*^ mutation (OARX: 50977717T > A, NC_019484) was genotyped by a RFLP analysis using the Mse1 restriction enzyme (New England Biolabs) after a first step of Terra PCR Direct Polymerase Mix amplification (Takara) using 1 μl sample of total blood. The accuracy of the *FecX*^*N*^ RFLP genotyping was validated by Sanger sequencing on few samples using the same amplification primers.

PCR amplifications were conducted independently for each locus studied on an ABI2400 thermocycler (Applied Biosystems) with the following conditions: 5 min at 94°C, 32 cycles of 30 s at the specific melting temperature, 30 s at 72°C and 30 s at 94°C, followed by 5 min at 72°C. The primers used in this study are listed in Supplementary Table S2.

### Whole-Genome Sequencing (WGS) Analysis

DNA sequencing libraries were constructed from 1 μg of genomic DNA using TruSeq DNA PCR-free Library Prep kit (Illumina). Sequencing was run on an Illumina HiSeq 2500 apparatus using a paired-end read length of 2 × 150 pb with the Illumina Reagent Kits as previously described (Demars et al., 2017). WGS was performed at the Genotoul-GeT core facility (INRA Toulouse)^2^. The raw reads of Illumina DNA sequencing were preprocessed by removing adapter sequences. After quality control, the FastQ files and metadata were submitted to the European Nucleotide Archive (ENA) at EMBL-EBI (accession number PRJEB35553). Reads mapping and variants calling were performed using the local instance of Galaxy^3^ at the Toulouse Midi-Pyrénées bioinformatics platform^4^. The cleaned paired reads were combined and mapped against the ovine genome assembly (Oar_v3.1.86) using BWA-MEM (Galaxy version 0.7.17.1). The resulting BAM files were sorted using Samtools_sort (Galaxy version 1.0.0). Sorted and indexed BAM files were visualized through the Integrative Genome Viewer, IGV software version 2.4.10 (Robinson et al., 2011). A GFF3 annotation file was obtained from Ensembl (Ovis_aries.Oar_V3.1.78). We applied GATK version 3.5-0 to perform SNP and InDel discovery and genotyping across the two samples simultaneously using standard filtering parameters according to GATK Best Practices recommendations (DePristo et al., 2011; Van der Auwera et al., 2013). Variants effect and annotation were realized by SNPEff version 4.1, and filtering of interesting variants was performed using the SNPSift tool.

### *In vitro* Transcription and Translation of BMP15

The full-length cDNA of ovine BMP15 [1480 bp (-297, + 1183) referring to ATG start codon] with or without the *FecX*^*N*^ mutation in position -290 was generated from oocyte-derived RNA after a reverse transcription (RT) step (described in RNA extraction and RT paragraph, primers are listed in Supplementary Table S2). The resulting PCR products were inserted by TA cloning into pGEM-T Easy plasmid (Promega) possessing T7 and SP6 promoters. The orientation of insertion and exclusion of unexpected PCR-induced mutations were controlled by Sanger sequencing.

*In vitro* transcription and translation were realized from 500 ng of cDNA pGEM-T construct using the TnT T7 Quick Coupled Transcription/Translation kit (Promega) and Transcend Biotin-Lysine-tRNA following the manufacturer’s protocol. Reactions for each construct were run in duplicate in six independent TnT experiments. The resulting BMP15 protein was detected using a Transcend non-radioactive translation detection system with chemiluminescent method (Promega) after reducing SDS-PAGE on a gradient (4–15%) polyacrylamide gel (Promega) and transfer onto nitrocellulose membrane. Chemiluminescent signal was capture by a ChemiDoc MP imaging system and images were analyzed with the Image Lab Software (Bio-Rad).

### BMP15 Promoter Activity

The promoter sequence of the ovine *BMP15* gene was amplified by PCR on genomic DNA from both homozygous *FecX*^+^ and homozygous *FecX*^*N*^ ewes. Two sizes of fragments were generated for cloning the *BMP15* promoter in front of the luciferase (Luc) reporter gene, a long (lg) form of 732 bp [(-743, -11) bp referring to ATG start codon] and a short (sh) form of 341bp [(-443, -102) bp]. The PCR products were engineered for digestion using *Kpn*I and *Hin*dIII restriction enzymes (New England Biolabs) and inserted into the pGL4.23 vector (Promega). The four resulting constructs (lgBMP15^+^-Luc, lgBMP15^*N*^-Luc, shBMP15^+^-Luc, shBMP15^*N*^-Luc) were controlled by Sanger sequencing. Primers used to generate these constructs are listed in Supplementary Table S2. Twenty-four hours after seeding (3.10^4^ cells/well, 24 wells plate), CHO (Chinese hamster ovary) cells were transfected using Lipofectamine 3000 (Invitrogen) with 500 ng/well of pGL4.23 constructs either empty or containing *BMP15* promoter fragment. Forty-eight hours after transfection, cells were lysed and assayed for luciferase activity (Luciferase reporter assay kit, Promega). Luminescence in relative light units (RLU) was measured by a Glomax microplate reader (Promega). Each construct was assayed in triplicate in six independent transfection experiments.

### RNA Extraction, Reverse Transcription, and Quantitative PCR

Total RNA from pools of five oocytes were extracted using the Nucleospin RNA XS kit according to the manufacturer’s protocol (Macherey–Nagel) and including a DNase1 treatment. The low quantity of RNA recovered did not allow quantification. So the equivalent of 1.25 oocyte was reverse-transcribed using SuperScript II reverse transcriptase (Invitrogen) and anchored oligo(dT)22 primer (1 μl at 10 μM). Primer design using Beacon designer 8.20 (Premier Biosoft), SYBR green real-time PCR cycling conditions using a QuantStudio 6 Flex Real-Time PCR system (Thermo Fisher Scientific) and amplification efficiency calculation [E = e^(–1/slope)^] were as previously described in Talebi et al. (2018a). Primer sequences, amplicon length, and amplification efficiency are listed in Supplementary Table S2. RNA transcript abundance was quantified using the ΔCt method with the mean expression of *GAPDH* and *SDHA* as internal references and following the formula R = [E_ref_^Ct ref^/E_target_^Ct target^]. The two reference genes were validated by the Bestkeeper algorithm (Pfaffl et al., 2004).

### Data Analysis

Single-marker association analyses were conducted using a Fisher’s exact test, and a Bonferroni correction has been applied to check for significance levels. The chromosome- and genome-wide values have been established as mentioned by Balding (2006). Statistical analyses were done using PLINK1.9 software under a case/control design (Chang et al., 2015). Among the 79 data sets of 47,446 SNPs analyzed, the LS trait was considered as the case when mean LS ≥ 2.18 (*n* = 39) and control when LS ≤ 1.45 (*n* = 40). Haplotypic association analysis on the X chromosome were performed using FastPhase software (Scheet and Stephens, 2006). Empirical significance levels were calculated using maximum statistic permutation approach [max (T), *n* = 1000].

Allele effect on LS was estimated in NV and BMC breeds on data extracted from the French national database for genetic evaluation and research managed by the Institut de l’Elevage (French Livestock Production Institute) and the CTIG (Centre de Traitement de l’Information Génétique, Jouy-en-Josas, France). Only females born after 2000 were retained (27,754 NV ewes with 122,110 LS records and 110,848 BMC ewes with 461,405 LS records) with their pedigree over five generations. The *FecX*^*N*^ genotype effect on the subset of 79 case/control animals was assessed by one-way ANOVA, followed by the Newman–Keuls *post hoc* test. For the large animal cohort analyses, the linear mixed models used were as similar as possible to those of the national genetic evaluation system (Poivey et al., 1995). In the present study, the following fixed effects were considered: (i) the genotype at the *FecX* locus; (ii) the month of birth (12 levels); (iii) a physiological status effect combining parity, age at first lambing, rearing mode, and postpartum interval (44 levels); and (iv) a combination of the flock year and season effect. Two random effects were added to the model: a permanent environmental effect and an animal additive genetic effect. Moreover, an additional fixed effect of the reproduction type was considered for the BMC breed for which some hormonal treatments are used each year (87 and 13% for natural and induced estrus, respectively). For the NV breed, because the *FecL*^*L*^ allele is also segregating in the population (Chantepie et al., 2018), the effect of the genotype at this locus (2252 known and 25,502 unknown genotypes) as well as its interaction with the genotypes at the *FecX* locus were considered. All these models were fitted using the ASReml software (Gilmour et al., 2009).

The comparison between *FecX* alleles for BMP15 protein quantification was analyzed using Student’s *t*-test, using Welch’s correction for unequal variance. For reporter luciferase assays, differences between constructs were analyzed by one-way ANOVA followed by Newman–Keuls *post hoc* test. QPCR data for *BMP15* and *GDF9* expression in oocytes were analyzed by two-way ANOVA considering genotype and breed effects. *P* < 0.05 was the threshold for statistical significance. All these experimental data are presented as means ± *SEM* and were analyzed using Prism 6 (GraphPad Software Inc.).

## Results

### Genetic Association Analyses

**FIGURE 1:**
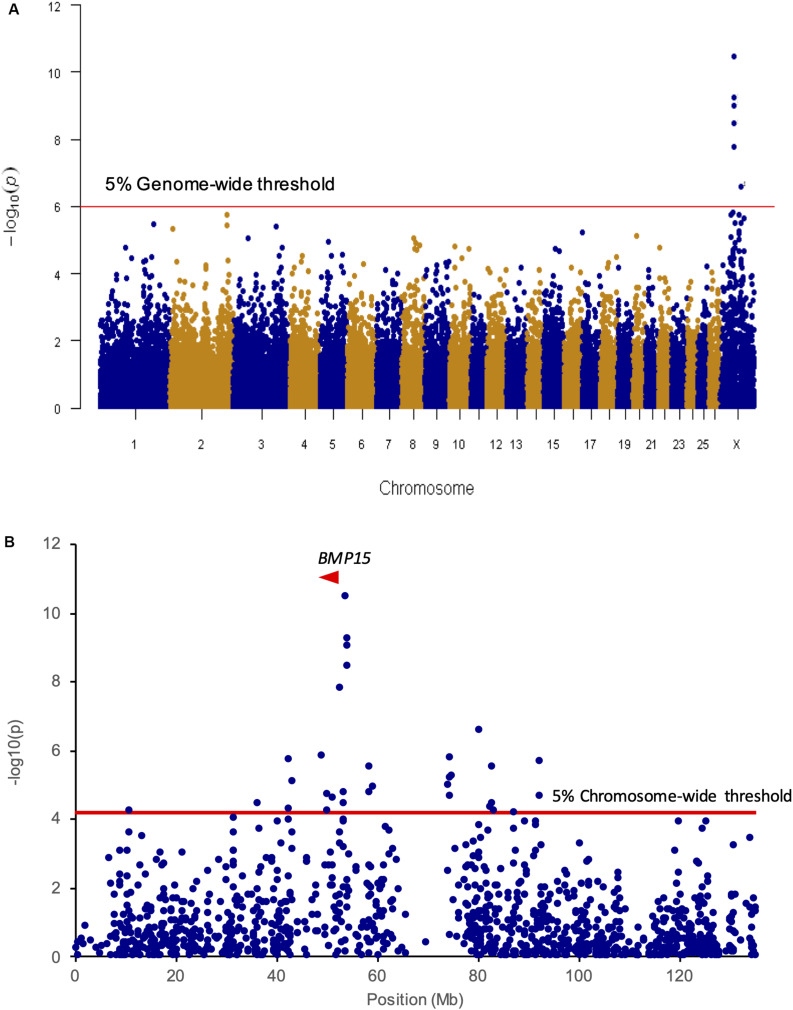
Genome- and chromosome-wide association results. **(A)** Genome-wide association results for litter size in the NV sheep population. Manhattan plot shows the combined association signals [-log_10_(*p-*value)] on the *y*-axis vs. SNPs position in the sheep genome on the *x*-axis and ordered by chromosome number (assembly OARv3.1). Red line represents the 5% genome-wide threshold. **(B)** OARX chromosome-wide association results. The curve shows the combined association signals [-log_10_(*p-*value)] on the *y*-axis vs. SNPs position on the X chromosome on the *x*-axis (assembly OARv3.1). Red line represents the 5% chromosome-wide threshold. The *BMP15* gene location is indicated by a red arrowhead.

**FIGURE 2:**
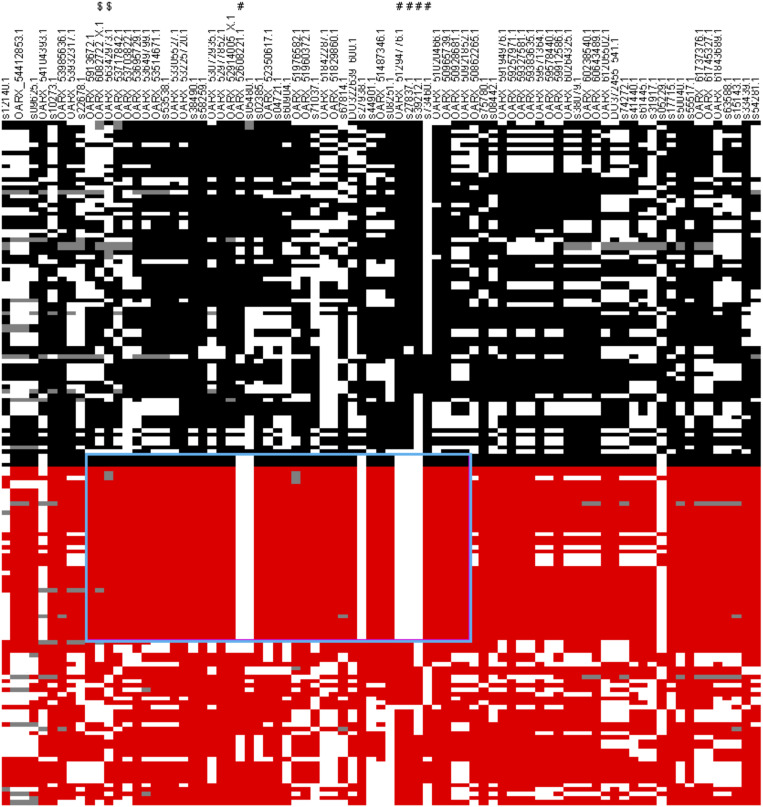
Clusterization of haplotypes reconstructed at the OARX locus. Eighty markers encompassing the OARX region of interest (50.6–54.1 Mb) were selected to construct haplotypes from 39 cases and 40 control animals. Each column represents one SNP, and each line represents one haplotype. For one marker (i) allele 1 is in black in controls or in red in cases, (ii) allele 2 is in white when the phase was unambiguous, and (iii) gray color represents unphased SNP. Haplotypes were ordered to distinguish controls vs. cases and clusterized to classify similar clades of haplotypes. The # sign flags SNP significantly associated with LS at genome-wide level. The $ sign flags SNP flanking the *BMP15* genes (50,970,938–50,977,454 bp). The specific haplotype preferentially selected in highly prolific ewes (cases) is symbolized by the cyan rectangle.

A first set of genomic DNA from 30 NV ewes without the *FecL*^*L*^ prolific allele at the *B4GALNT2* locus (LS records ranging from 2.00 to 3.00) was genotyped for already known mutations affecting sheep prolificacy at the three other loci, *BMPR1B*, *GDF9*, and *BMP15*. Using a specific RFLP assay (*BMPR1B*, Wilson et al., 2001) or Sanger sequencing of coding parts (*GDF9* and *BMP15*, Talebi et al., 2018b), none of the known mutations was evidenced (data not shown). Thus, to establish the genetic determinism of the remaining LS variation in this population, 80 ewes were genotyped by an Illumina Ovine SNP50 Genotyping Beadchip. The allele frequencies of the most highly prolific ewes (cases, *n* = 40, mean LS = 2.47) and lowly prolific ewes (controls, *n* = 40, mean LS = 1.23) were compared to identify loci associated with LS using GWAS according to the procedures described in section “Materials and Methods.” Finally, genotype data were obtained from 79 animals (39 cases, 40 controls). Six markers located on OARX were significantly associated with LS variation at the genome-wide level after Bonferroni correction (Figure 1A and Table 1). Importantly, at the chromosome-wide level, a cluster of 26 significant markers encompassed the location of the *BMP15* candidate gene (Figure 1B). In order to better characterize this locus on the X chromosome, we have determined, for each individual, the most likely linkage phase across 80 markers (10 Mb) including the significant region. After haplotype clusterization, a specific segment of 3.5 Mb (50,639,087–54,114,793 bp, OARv3.1 genome assembly) was identified to be more frequent in highly prolific cases than in controls (f_cases_ = 0.51 vs. f_controls_ = 0.037, *P* = 1.92E^–11^, Chi-square test) (Figure 2). This identified segment contained the *BMP15* gene (50,970,938–50,977,454 bp, OARv3.1) well known to play a crucial role in ovarian function and to be a target of numerous mutations in its coding region controlling prolificacy (Persani et al., 2014).

**TABLE 1:** Markers significantly associated with litter size.

**SNP**	**Chromosome**	**Position^a^**	**MAF^b^**	***P*_Unadj_^c^**	***P*_Chrom_^d^**	***P*_Genome_^e^**
OARX_51294776.1	OARX	53,756,339	0.28	3.52E^–11^	4.18E^–08^	1.67E^–06^
s27837.1	OARX	53,825,247	0.30	5.83E^–10^	6.92E^–07^	2.77E^–05^
s73460.1	OARX	53,905,939	0.44	9.59E^–10^	1.14E^–06^	4.55E^–05^
s39212.1	OARX	53,852,735	0.44	3.46E^–09^	4.11E^–06^	1.64E^–04^
OARX_52608221.1	OARX	52,367,253	0.41	1.68E^–08^	2.00E^–05^	7.99E^–04^
s46003.1	OARX	80,222,479	0.44	2.56E^–07^	3.03E^–04^	1.21E^–02^
OARX_55032299.1	OARX	48,942,926	0.30	1.57E^–06^	1.86E^–03^	NS
OARX_72164491.1	OARX	74,388,397	0.23	1.71E^–06^	2.03E^–03^	NS
OARX_49135019.1	OARX	42,475,099	0.35	1.80E^–06^	2.13E^–03^	NS
OARX_111306030.1	OARX	920,41,520	0.27	2.25E^–06^	2.67E^–03^	NS
OARX_102620828.1	OARX	82,796,975	0.41	3.02E^–06^	3.59E^–03^	NS
s31917.1	OARX	58,202,482	0.44	3.12E^–06^	3.70E^–03^	NS
OARX_72351736.1	OARX	74,590,448	0.19	5.63E^–06^	6.68E^–03^	NS
OARX_72263548.1	OARX	74,498,463	0.17	6.45E^–06^	7.66E^–03^	NS
OARX_49564109.1	OARX	42,876,169	0.49	8.16E^–06^	9.69E^–03^	NS
DU400878_520.1	OARX	73,847,207	0.27	9.96E^–06^	1.18E^–02^	NS
s54281.1	OARX	58,993,959	0.24	1.20E^–05^	1.42E^–02^	NS
s05229.1	OARX	58,346,644	0.46	1.71E^–05^	2.03E^–02^	NS
s27938.1	OARX	53,275,559	0.35	1.77E^–05^	2.10E^–02^	NS
OARX_54104393.1	OARX	49,870,983	0.36	2.03E^–05^	2.41E^–02^	NS
OARX_72236232.1	OARX	74,464,263	0.18	2.23E^–05^	2.64E^–02^	NS
OARX_111349974.1	OARX	92,085,118	0.18	2.23E^–05^	2.65E^–02^	NS
OARX_53703822.1	OARX	51,193,144	0.35	2.59E^–05^	3.08E^–02^	NS
OARX_43227227.1	OARX	36,235,514	0.32	3.41E^–05^	4.04E^–02^	NS
OARX_51842287.1	OARX	53,162,079	0.49	3.43E^–05^	4.08E^–02^	NS
OARX_102654502.1	OARX	82,837,158	0.20	3.65E^–05^	4.33E^–02^	NS

*^*a*^Position of markers are based on the OARv3.1 assembly in bp. ^*b*^MAF, minor allele frequency. ^*c*^P_*Unadj*_ corresponds to exact unadjusted p-value for the Fisher’s test. ^*d*^P_*Chrom*_ corresponds to p-value after chromosome-wide Bonferroni correction. ^*e*^P_*Genome*_ corresponds to p-value after genome-wide Bonferroni correction (NS, non-significant).*

### Characterization of the Mutation

Although the *BMP15* gene could be considered as a positional and functional candidate gene, no mutation was evidenced by Sanger sequencing of the *BMP15* coding regions of the most prolific ewes studied. In order to find the potential causal mutation, we sequenced the whole genome of two specifically chosen ewes based on the shortest haplotype within the region (homozygous reference vs. homozygous variant) and their opposite extreme phenotypes (LS 1.1 vs. 2.8).

**FIGURE 3:**
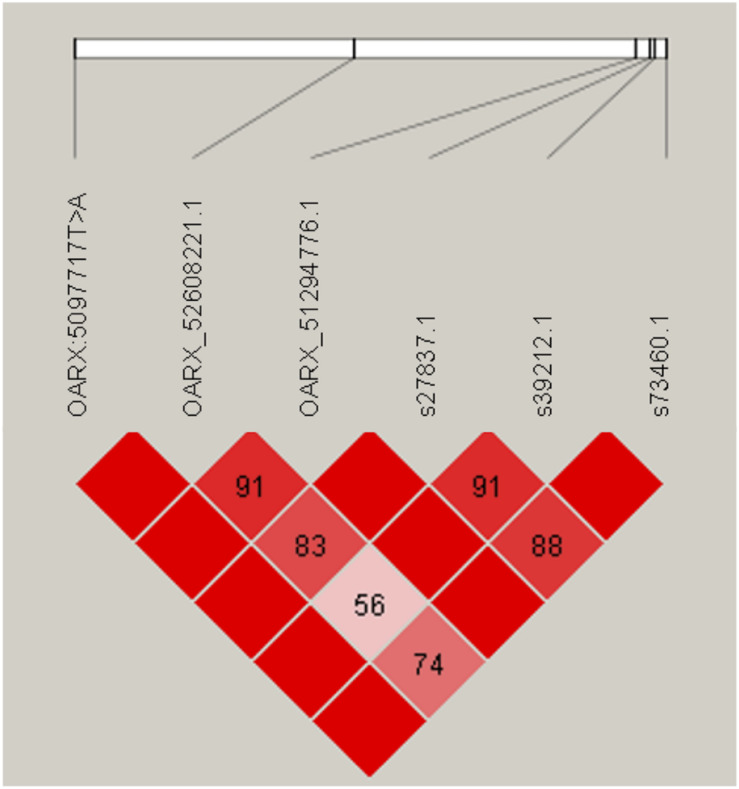
Linkage disequilibrium (LD) plot. Generated by Haploview, pairwise LD between SNP markers (OARX: 50977717T > A and the five genome-wide significant LS-associated markers) is represented by the D’ value. Strong LD is represented in dark red boxes (D’ = 100) and weaker LD (D’ < 100) in lighter red boxes.

Variant search analysis and annotations through the GATK toolkit was limited to the OARX: 50,639,087–54,114,793 bp region. We detected 60 SNPs and 90 small insertions and deletions (INDELs) with quality score > 30 (Supplementary Table S1). Among them, we particularly focused on the 85 variants located within annotated genes (upstream, exon, intron, splice acceptor or donor, and downstream localization). After filtering these 85 variants for allele sharing with other breeds based on SheepGenome DB^5^ and 68 publicly available domestic sheep genomes (International Sheep Genomics Consortium)^6^, none of them were removed, all being NV breed-specific. Finally, and based on prolificacy gene knowledge, we were particularly interested in one SNP (T > A) identified in the upstream region of the *BMP15* gene at position 50,977,717 bp on OARX v3.1. We then developed an RFLP assay to specifically genotype for this polymorphism. Among the 79 animals of the GWAS, 31 ewes were heterozygous and six homozygous for the A variant allele. As shown in Table 2, most of the A carrier ewes were in the highly prolific Case group (34 among 39), and only three set in the Control group. When associating the LS performance of the 79 ewes to their genotype at the OARX: 50977717T > A SNP, the A non-carriers exhibited a mean LS of 1.36, heterozygous T/A a mean LS of 2.32, and homozygous A/A a mean LS of 2.73, indicating that the A allele of this polymorphism was strongly associated with increased LS in NV (T/A or A/A vs. T/T, *P* < 1E^–3^, one-way ANOVA). Furthermore, this polymorphism appears to be in total linkage disequilibrium with the six more significant markers from the GWAS analysis (Figure 3). Genotype information at the OARX: 50977717T > A locus was introduced in the GWAS analysis. This SNP appeared as the most significant marker associated with the prolificacy phenotype (*P*_unadjusted_ = 1.93E^–11^, *P*_Chromosome–wide corrected_ = 1.62E^–14^ and *P*_Genome–wide corrected_ = 9.13E^–07^), suggesting that it could be the causal mutation (Supplementary Figure S1). In accordance with the *Fec* gene nomenclature, the mutant allele identified upstream of the *BMP15* gene in NV sheep was named *FecX*^*N*^.

**TABLE 2:** Distribution of OARX:50977717T > A SNP genotypes and associated LS in case and control groups.

**Group**	**Genotype**	**TT**	**TA**	**AA**
Low prolific control	n	37	3	
	Raw mean LS	1.22	1.39	
Highly prolific case	n	5	28	6
	Raw mean LS	2.41	2.43	2.73
Total		42	31	6

As described for other prolific alleles, such as *FecB*^*B*^, *FecX*^*G*^, *FecG*^*H*^, *FecX*^*Gr*^, and *FecL*^*L*^, a given mutation can segregate in several sheep populations (Davis et al., 2002; Mullen et al., 2013; Chantepie et al., 2018; Ben Jemaa et al., 2019). We have tested the *FecX*^*N*^ allele presence in a diversity of 26 sheep breeds representing 725 animals (Rochus et al., 2018). Among the breeds tested, the *FecX*^*N*^ genotyping has confirmed the segregation of this mutation in NV breed and revealed its presence in the Blanche du Massif Central (BMC) and Lacaune breeds (Table 3). Additionally, the *FecX*^*N*^ variant was absent from the Ensembl variant database^7^ compiling information from (i) dbSNP, (ii) whole-genome sequencing information from the NextGen project (180 animals from various Iranian and Moroccan breeds), and (iii) the International Sheep Genome Consortium (551 animals from 39 breeds all over the world).

**TABLE 3:** *FecX*^*N*^ genotype distribution from a diversity panel of French ovine breeds.

		**Genotype^*a*^**				**Genotype^*a*^**	
**Breed**	**Total**	**+/+ (+*/Y*)**	***N/*+ (*N/Y*)**	**Breed**	**Total**	** + /+ (+*/Y*)**	***N/*+ (*N/Y*)**
Berrichon du Cher	29	29		Mourerous	26	26	
Blanche Massif Central	31	27	4	Mouton Vendéen	30	30	
Causse du Lot	32	32		Noire du Velay	28	26	2
Charmoise	31	31		Préalpes du sud	27	27	
Charollais	29	29		Rava	29	29	
Corse	30	30		Romane	29	29	
Ile de France	28	28		Romanov	26	26	
Lacaune (meat)	42	40	2	Rouge de l’Ouest	28	28	
Lacaune (dairy)	40	40		Roussin	30	30	
Limousine	30	30		Suffolk	20	20	
Manech tête rousse	29	29		Tarasconnaise	32	32	
Martinik	22	22		Texel	21	21	
Merinos d’Arles	26	26					
				Total	725	717	8

*^*a*^+/+ or N/+ females, N/Y or N/+ hemizygous males.*

### *FecX*^*N*^ Genotype Frequency and Effect on Prolificacy

Large cohorts of ewes, chosen at random, were genotyped in order to accurately estimate the allele frequencies in the NV and the BMC populations (Table 4). The frequency of the N prolific allele at the *FecX* locus was similar in both populations, 0.11 and 0.10, with a distribution of 19.4 and 17.6% heterozygous, 1.5 and 1% homozygous carriers in NV and BMC, respectively. The genotype frequencies were consistent with the Hardy–Weinberg equilibrium (HWE) in both breeds (NV *P* = 0.28 and BMC *P* = 0.76).

**TABLE 4:** *FecX*^*N*^ frequencies and effects on LS in NV and BMC breeds.

	**Breed**						
	**NV (*n* = 2323)**				**BMC (*n* = 2456)**		
***FecX* genotype**	**+/+**	***N/*+**	***N/N***		**+/+**	***N/*+**	***N/N***
Number of animals	1839	450	34		1999	432	25
Frequency (%)	79.2	19.4	1.5		81.3	17.6	1
Raw mean LS	1.66	1.93	2.45		1.56	1.79	1.87
**ANOVA solutions^*a*^**							
*FecL* genotype +/+	0.00	0.22 (0.02)	0.65 (0.08)	NE	0.00	0.18 (0.02)	0.30 (0.09)
*L/*+	0.41 (0.02)	0.58 (0.04)	0.56 (0.16)	IE	0.23 (0.01)	0.35 (0.03)	0.17 (0.11)
*L/L*	0.72 (0.08)						

*^*a*^Estimated increased LS (± standard error) compared to L and N non-carriers from the linear mixed models with interaction between genotypes at FecX and FecL loci for Noire du Velay (NV) and between FecX genotypes and estrus type (NE, natural estrus; IE, Induced estrus) for Blanche du Massif Central (BMC). Global effects for genotypes, estrus type and their interactions were all significant (P < 0.001).*

**FIGURE 4:**
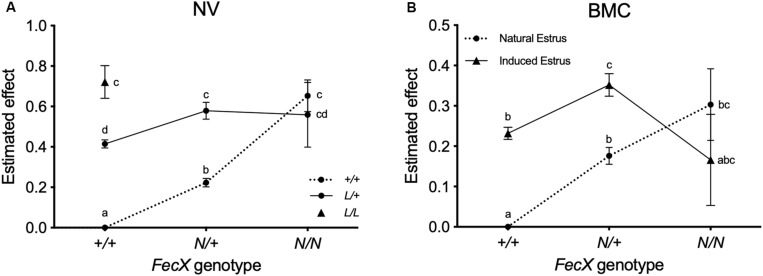
Estimated effect of *FecX*^*N*^. **(A)** Estimated effect of *FecX*^*N*^ knowing *FecL*^*L*^ genotype in Noire du Velay (NV). **(B)** Estimated effect of *FecX*^*N*^ according to induced or natural estrus in Blanche du Massif Central (BMC). Estimated increased LS and the corresponding standard errors from the linear mixed models with interaction between genotypes at the *FecX* and *FecL* loci for NV, and between *FecX* genotype and estrus type for BMC are given relative to *L* and *N* alleles non-carrier ewes (+/+). Different letters indicate significant differences (*P* < 0.01).

Based on the raw mean LS observations, the *FecX*^*N*^ carrier ewes clearly exhibited increased LS compared to non-carriers in both populations (Table 4). The L prolific allele at the *FecL* locus is also segregating in NV (Chantepie et al., 2018). Results of the linear mixed model showed that, for the NV breed, one copy of the *FecX*^*N*^ allele significantly increased LS by + 0.22, and two copies increased LS by +0.65, and a single copy of the *FecL*^*L*^ allele increased LS by + 0.41, and two copies by + 0.72. Based on the 80 ewes genotyped heterozygous at both loci, it appeared that the effects of *FecX*^*N*^ and *FecL*^*L*^ on LS were not fully additive, the expected LS being significantly slightly reduced by -0.05 (0.58 instead of 0.63) due to a significant interaction between the two loci (*P* < 0.001) (Figure 4A). For the BMC population, compared to *FecX^+^/FecX^+^* ewes, *FecX^*N*^/FecX^+^* exhibited increased LS by + 0.18 and *FecX^*N*^/FecX^*N*^* by + 0.30 under natural estrus (Figure 4B). The use of PMSG for estrus synchronization increased LS significantly among *FecX^+^/FecX^+^* ewes (+ 0.23) and *FecX^*N*^/FecX^+^* ewes (+ 0.18) while the effect on *FecX^*N*^/FecX^*N*^* ewes was negative (-0.13). The combined effect of the first copy of the *FecX*^*N*^ allele and the use of PMSG treatment was not fully additive, the interaction being significant (*P* < 0.001) although low (0.35 instead of 0.41) (Figure 4B).

### Functional Effects of the *FecX*^*N*^ Mutation

**FIGURE 5:**
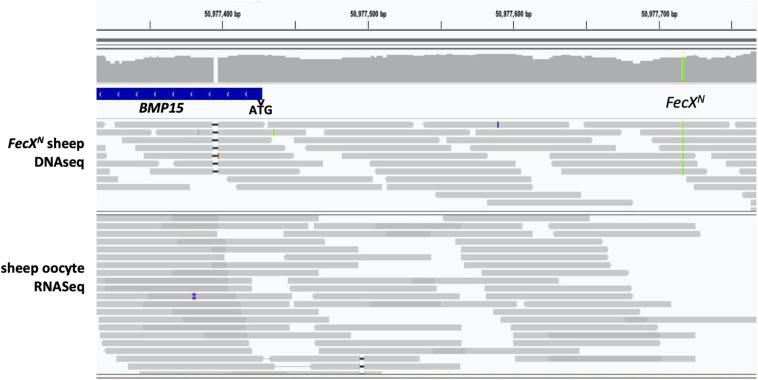
Localization of *FecX*^*N*^ in the *BMP15* upstream region. Integrative Genomics Viewer (IGV v2.4.10) snapshot of the ovine *BMP15* gene upstream region alignments (coordinates from OAR v3.1 assembly) with reads from whole genome DNA sequencing (DNASeq) of a homozygous *FecX*^*N*^ carrier ewe (green line) and total mRNA sequencing of ovine oocytes from small antral follicles (RNASeq, Bonnet et al., 2013), indicating a possible localization of *FecX*^*N*^ in the 5′UTR region of *BMP15* (ATG start codon is indicated by an arrowhead and *BMP15* gene annotation is on the minus strand).

**FIGURE 6:**
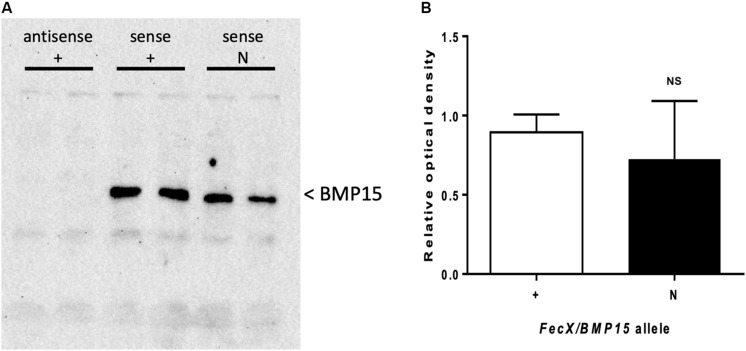
Effect of *FecX*^*N*^ mutation on the ovine BMP15 protein produced *in vitro. In vitro* transcription and translation were realized in duplicate in six independent experiments from antisense *BMP15* cDNA carrying the wild-type allele (antisense +) as negative control or sense *BMP15* cDNA with (sense N) or without *FecX*^*N*^ (sense +). **(A)** A representative western blot experiment revealing the BMP15 protein. **(B)** Chemiluminescent signal of BMP15 was captured by a ChemiDoc MP imaging system and images were quantified (relative to sense +) and analyzed with the Image Lab Software (Bio-Rad). Means (± *SEM*) were not significantly different (NS) after Student’s *T-*test with Welch’s correction.

As described above, *FecX*^*N*^ is located upstream of the coding region of the *BMP15* gene when referencing to the ovine genome v3.1 (ensembl.org) or v4.0 (ncbi.nlm.nih.gov). In both versions of the ovine genome, the *BMP15* gene annotation begins at the ATG start site and *FecX*^*N*^ is located 290 pb upstream, possibly in the 5′UTR and/or the proximal promoter region. As a first approach, we took advantage of RNA sequencing data from ovine oocytes publicly available at EMBL-EBI (Bonnet et al., 2013). After reads mapping against the ovine genome (v3.1) using STAR2 aligner within the Galaxy pipeline and visualization with Integrative Genome Viewer (IGV), Figure 5 shows the location of *FecX*^*N*^ within the possible 5′UTR of the *BMP15* gene when expressed in the oocyte. Consequently, we have first tested the potential functional impact of *FecX*^*N*^ on the *in vitro* stability and translatability of the *BMP15* mRNA. Thus, the reference (T, *FecX*^+^) and variant (A, *FecX*^*N*^) forms of the ovine BMP15 cDNA (-297, +1183 referring to ATG start codon) were cloned in a pGEM-T vector for subsequent *in vitro* T7 promoter-dependent transcription/translation experiment using reticulocyte lysate solution. As shown in Figure 6, the Western blotting of the BMP15 proteins produced from both forms, and their chemiluminescent quantification revealed that the *FecX*^*N*^ mutation had no significant impact on the overall stability and translatability of the *BMP15* mRNA in this condition.

**FIGURE 7:**
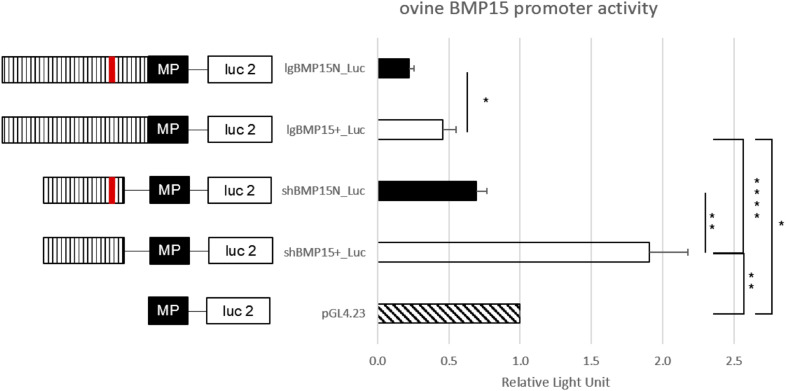
Functional effect of *FecX*^*N*^ mutation on the *BMP15* promoter activity. *In vitro* reporter luciferase assay from CHO cells transiently transfected with empty vector or wild-type *BMP15* promoter (BMP15^+^) or mutant *BMP15* promoter (BMP15^*N*^). Two fragments were generated, a long (lg) form of 732 bp (-743, -11 bp referring to ATG start codon) and a short (sh) form of 341 bp (-443, -102 bp). Results are expressed as means ± *SEM* of the relative light unit (RLU) from six independent transfection experiments in triplicate. Asterisk indicates significant difference after one-way ANOVA *P < 0.05; **P < 0.01; ****P < 0.0001. MP, minimal promotor of pGL4.23; Luc 2, luciferase reporter gene; hatched bar, *BMP15* promotor; red line, *FecX*^*N*^ mutation.

**FIGURE 8:**
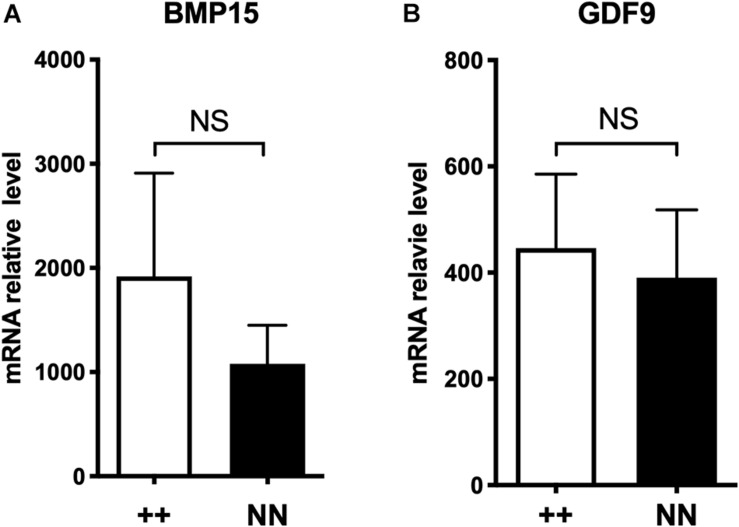
Effect of *FecX*^*N*^ mutation on *BMP15* and *GDF9* expression in ovine oocyte. Quantitative real-time PCR results of *BMP15* **(A)** and *GDF9* **(B)** expression in oocyte pools from growing (1–3 mm) follicles (two pools of five oocytes per animal) from homozygous *FecX*^*N*^ carrier (*N/N*, *n* = 5) and non-carrier ewes (+/+, *n* = 5) during the follicular phase of the estrus cycle. Results are expressed as means ± *SEM* of the mRNA relative level for each genotype, using *GAPDH* and *SDHA* as internal references. Raw data were analyzed by two-way ANOVA. Means (± SEM) were not significantly different (NS).

As a second hypothesis, we have tested the *FecX*^*N*^ impact on the *BMP15* promoter activity. Two promoter regions were tested [(-743, -11) bp and (-443, -102) bp referring to ATG start codon] cloned in front of the luciferase reporter gene and transiently expressed in CHO cells cultured *in vitro*. As shown by the luciferase assays (Figure 7), the *FecX*^*N*^ variant was able to significantly reduce the luciferase activity in the context of both the long or the short BMP15 promoters, indicating the possible inhibitory impact of *FecX*^*N*^ on *BMP15* gene expression. To go further with this hypothesis, *in vivo* BMP15 gene expression was measured directly on isolated oocytes pools from NV and BMC homozygous ewe carriers and non-carriers of the *FecX*^*N*^ allele. Even if the mean expression of *BMP15* checked by real-time qPCR was decreased by twofold in the oocytes of *FecX*^*N*^ carriers, this difference appeared as not significant (*P* = 0.166, genotype effect, two-way ANOVA) due to a large variability between animals. In parallel, the mean expression of the second oocyte-specific prolificacy major gene *GDF9* seemed unaffected by the presence of *FecX*^*N*^ (Figure 8).

## Discussion

The present study identified the g.50977717T > A variant on the ovine chromosome X upstream of the *BMP15* gene as the most likely causative mutation for the increased prolificacy of the NV ewes. The highly significant genetic association with the extreme LS phenotype, the significant effect of the A variant on increasing prolificacy by + 0.2 lamb per lambing in a large set of NV ewes, also found in the BMC genetic background, and the demonstrated action on *BMP15* transcriptional activity all support the causality of this mutation named *FecX*^*N*^.

The *BMP15* gene is at the top of the list of candidate genes controlling ovarian function, ovulation rate, and thus prolificacy in the ovine species with 10 independent mutations identified out of the 17 already known. Indeed, eight SNPs and two small INDELs, all within the open reading frame, were evidenced affecting prolificacy and BMP15 function. Among these mutations, two SNPs and the two INDELS impaired the protein production either by generating premature stop codon (*FecX*^*H*^, Galloway et al., 2000; *FecX*^*G*^, Hanrahan et al., 2004) or by breaking the reading frame (*FecX*^*R*^, Martinez-Royo et al., 2008, *FecX*^*Bar*^, Lassoued et al., 2017). The six other SNPs generate non-conservative amino acid substitutions, one being not functionally tested (L252P, Amini et al., 2018), and all others leading to a loss of function of BMP15 ranging from inhibited protein production (*FecX*^*L*^, Bodin et al., 2007) and impaired interaction with GDF9 (*FecX*^*I*^ and *FecX*^*B*^, Liao et al., 2004) to altered cell signaling activity (*FecX*^*Gr*^ and *FecX*^*O*^, Demars et al., 2013). In contrast with the 10 mutations described above, the *FecX*^*N*^ variant evidenced in the present study is not located in the open reading frame of *BMP15* and does not alter the protein sequence. However, no other polymorphism genetically linked to *FecX*^*N*^ was found in the *BMP15* coding sequence when checked by whole genome or local Sanger sequencing of the *BMP15* gene from *FecX*^*N*^ carrier animals. Of course, this does not rule out the possibility of a polymorphism lying in another gene nearby with a still unknown role in the ovarian function and prolificacy. Nevertheless, we did not find any polymorphism (SNP and INDEL) altering the coding sequence of genes annotated in the significantly LS-associated genetic region of 3.5 Mb on OARX (Supplementary Table S1), leaving *BMP15* as the most obvious candidate.

Whatever the version of the ovine reference genome (Oar_v3.1, Oar_v4.0, or even the last Oar_rambouillet_v1.0), the annotation of the *BMP15* gene always starts at the ATG-initiating codon. Using publicly available transcriptome data from ovine oocytes RNAseq analysis, we were able to show that *FecX*^*N*^ located 290 bp upstream of *BMP15* could stand in its 5′UTR region. From our *in vitro* functional analyses, *FecX*^*N*^ was not demonstrated to influence the translatability of the *BMP15* mRNA, but on the contrary, it was shown to decrease the *BMP15* promoter activity. Little is known about transcription factors able to regulate *BMP15* expression. Several regulatory elements were evidenced in pig *BMP15* promoter hosting consensus-binding sites for LHX8, NOBOX, and PITX1 transcription factors. However, only LHX8 was demonstrated as functionally activating the porcine BMP15 promoter activity (Wan et al., 2015). In human, a regulatory mutation in the 5′UTR of *BMP15* (c.-9C > G) was associated to non-syndromic premature ovarian failure (Dixit et al., 2006), and also iatrogenic ovarian hyperstimulation syndrome (Morón et al., 2006). This mutation was shown to enhance the fixation of the PITX1 factor transactivating the BMP15 promoter (Fonseca et al., 2014). However, the *FecX*^*N*^ position does not fit with the syntenic location of porcine LHX8 and human PITX1 binding sites on the ovine *BMP15* promoter. Using the MatInspector promoter analysis tool (Genomatix), we were only able to hypothesize an alteration by *FecX*^*N*^ of a putative TATA-box-like sequence (TTAAATA > TTATATA). Unfortunately, our electromobility shift assay attempts using CHO nuclear extracts failed to demonstrate the binding of any factor at the *FecX*^*N*^ position, preventing us from defining the precise molecular mechanism by which *FecX*^*N*^ decreases *BMP15* promoter activity.

The inhibition of the promoter activity suggests a transcriptional regulatory role of *FecX*^*N*^ on *BMP15* gene expression. That is the reason why *BMP15* mRNA accumulation in homozygous *FecX^*N*^/FecX^*N*^* oocytes was checked and compared to non-carrier oocytes. The twofold decrease of *BMP15* mean expression in oocytes of *FecX*^*N*^ carriers was not statistically significant. However, the moment we have chosen during the follicular phase of the late folliculogenesis for the comparative analysis between *FecX*^+^ and *FecX*^*N*^ oocytes from 1 to 3 mm antral follicles without controlling the atretic status might not be optimal to visualize a highly significant differential expression of *BMP15*. To this could be added an induced effect on oocyte by our experimental procedures, progestagen treatment for estrus cycle synchronization and/or oocyte denudation (Rose et al., 2013; Menchaca et al., 2018). The *BMP15* gene expression in ovine oocytes begins during the primary stage of follicular development, and its expression increases up to the antral stages where it stays stable (McNatty et al., 2005; Bonnet et al., 2011; Kona et al., 2016; Bonnet and Fabre, unpublished data). Moreover, the streak ovaries phenotype of infertile ewes carrying homozygous mutations in *BMP15* have evidenced its crucial role in controlling the primary to secondary follicle transition (Galloway et al., 2000; Bodin et al., 2007; Lassoued et al., 2017). Consequently, it would certainly be appropriate to follow the *BMP15* expression in *FecX*^*N*^ carrier ewes from these early stages of folliculogenesis to better decipher the mutation impact on ovarian physiology. Nevertheless, the fact that *FecX*^*N*^ could inhibit the *BMP15* gene expression fits well with the physiological and molecular models associating BMP system loss of function and increased sheep prolificacy (Fabre et al., 2006; Demars et al., 2013).

One copy of the *FecX*^*N*^ allele significantly increased by +0.30 to +0.50 the raw mean LS of NV ewes. When corrected for different environmental effects and more particularly for the genotype at the *FecL* locus, the estimated effect of *FecX*^*N*^ on LS was +0.22 lamb per lambing for the first copy and + 0.43 for the second copy. The *FecX*^*N*^ effect is in the range of already known prolific alleles in various sheep breeds (Jansson, 2014) and explains about 20% of the genetic variance of prolificacy without considering information at the *FecL* locus and up to 50% when polygenic effect is corrected for the presence of *FecL*^*L*^, confirming its status of major gene. The effect of *FecX*^*N*^ on LS seems relatively independent of the genetic background. Indeed, the estimated positive effect of the first copy of *FecX*^*N*^ on prolificacy in NV was confirmed in the BMC breed with + 0.18 lamb per lambing based on natural estrus. Moreover, the same robust effect was observed even in the presence of PMSG for synchronizing the estrus cycles preceding the lambing. The same observation is made for other mutations controlling sheep prolificacy. For instance, the *FecL*^*L*^ allele exhibited a similar effect on LS in NV (+0.41, present study; +0.42, Chantepie et al., 2018) Lacaune, +0.47 (Martin et al., 2014), and D’man (+0.30, Ben Jemaa et al., 2019), and this was also observed for the *FecB*^*B*^ allele introgressed in several populations (Kumar et al., 2008).

By genotyping a diversity panel, we also evidenced the presence of 2 *FecX*^*N*^ carrier animals in the Lacaune meat strain, which will require further genotyping of numerous animals. If this is confirmed, the Lacaune meat breed will be another population, as Belclare, in which three different natural prolific mutations are segregating (Hanrahan et al., 2004; Bodin et al., 2007; Drouilhet et al., 2013). The presence of *FecL*^*L*^ in both NV and Lacaune and the presence of *FecX*^*N*^ in NV, BMC, and Lacaune also raise the question of the origin of these mutations. From population structure analysis, it was shown that NV, BMC, and Lacaune shared the same origin within the European southern sheep populations that may explain the segregation of the same mutations in these populations (Rochus et al., 2018).

## Conclusion

In conclusion, through a case/control GWAS strategy and genome sequencing, we have identified in the NV breed a second prolific mutation named *FecX*^*N*^ affecting the expression of the *BMP15* gene, a well-known candidate gene controlling OR and LS in sheep. This work confirms the relevance of the whole genome approaches to decipher the genetic determinism of the prolificacy trait. Homozygous *FecX*^*N*^/*FecX*^*N*^ animals were still hyperprolific as previously observed for *FecX*^*Gr*^ and *FecX*^*O*^ but in contrast with sterile animals observed for the seven other *FecX* homozygous variants in *BMP15*. As an upstream regulatory mutation, *FecX*^*N*^ also contrasts with these nine other prolific causal mutations all evidenced in the coding part of *BMP15* and altering the protein function. Thanks to this new sheep model, the genetic etiology of ovarian pathologies in women could be improved by searching polymorphisms, not only in the coding region, but also in the regulatory parts driving the *BMP15* expression within the oocyte.

## Data Availability Statement

The datasets generated for this study can be found in the European Nucleotide Archive (ENA) at EMBL-EBI (accession number PRJEB35553).

## Ethics Statement

All experimental procedures were approved (approval number 01171.02) by the French Ministry of Teaching and Scientific Research and local ethical committee C2EA-115 (Science and Animal Health) in accordance with the European Union Directive 2010/63/EU on the protection of animals used for scientific purposes. Written informed consent for participation was not obtained from the owners because breeders in selection schemes are affiliated to selection organisms and give their tacit agreement for animal participation to these organisms collaborating with research institutes.

## Author Contributions

LC conducted the investigation, validation, formal analysis and visualization of the data, and wrote the original draft. LB participated in the conceptualization, formal analysis, data curation, and final writing, review, and editing of the manuscript. JS, FW, FP-P, JR, and LD participated in the investigation and getting the biological resources. LD helped in writing, review, and editing of the final manuscript. SF was implicated in the conceptualization, supervision, funding acquisition, investigation, formal analysis and writing, review, and editing of the final manuscript. All authors contributed to the article and approved the submitted version.

## Conflict of Interest

The authors declare that the research was conducted in the absence of any commercial or financial relationships that could be construed as a potential conflict of interest.

## Footnotes

1 www.labogena.fr

2 get.genotoul.fr

3 https://galaxyproject.org

4 http://sigenae-workbench.toulouse.inra.fr

5 http://sheepgenomesdb.org/

6 http://www.sheephapmap.org/

7 http://www.ensembl.org

## Supplementary Material

FIGURE S1 Genome-wide and chromosome-wide association results integrating the SNP OARX: 50977717T > A. **(A)** The SNP OARX: 50977717T > A genotypes were included in the SNP50 Beadchip data for genome-wide association analysis for litter size in the NV sheep population. Manhattan plot shows the combined association signals [-log_10_(*p-*value)] on the *y*-axis vs. SNPs position in the sheep genome on the *x*-axis and ordered by chromosome number (assembly OARv3.1). Red line represents the 5% genome-wide threshold. **(B)** OARX chromosome-wide association results. The curve shows the combined association signals [-log_10_(*p-*value)] on the *y*-axis vs. SNPs position on the X chromosome on the *x*-axis (assembly OARv3.1). Red line represents the 5% chromosome-wide threshold. In both panels, the position of the SNP OARX:50977717T > A is indicated by a red dot. In **(B)**, the *BMP15* gene location is indicated by a red arrowhead.

TABLE S1 List of variants found in the OARX: 50639087–54114793 region. Listing of 60 SNPs and 90 small INDELs with quality score > 30.

TABLE S2 List of primers used in the study. Locations of primers are based on the OARv3.1 ovine genome assembly available on ensembl.org.
