# Supplementary figures and images for: Genome-Wide Identification of a Regulatory Mutation in BMP15 Controlling Prolificacy in Sheep
Source: Front Genet. 2020 Jun 19;11:585. doi: 10.3389/fgene.2020.00585 (PMC7317000; doi:10.3389/fgene.2020.00585)

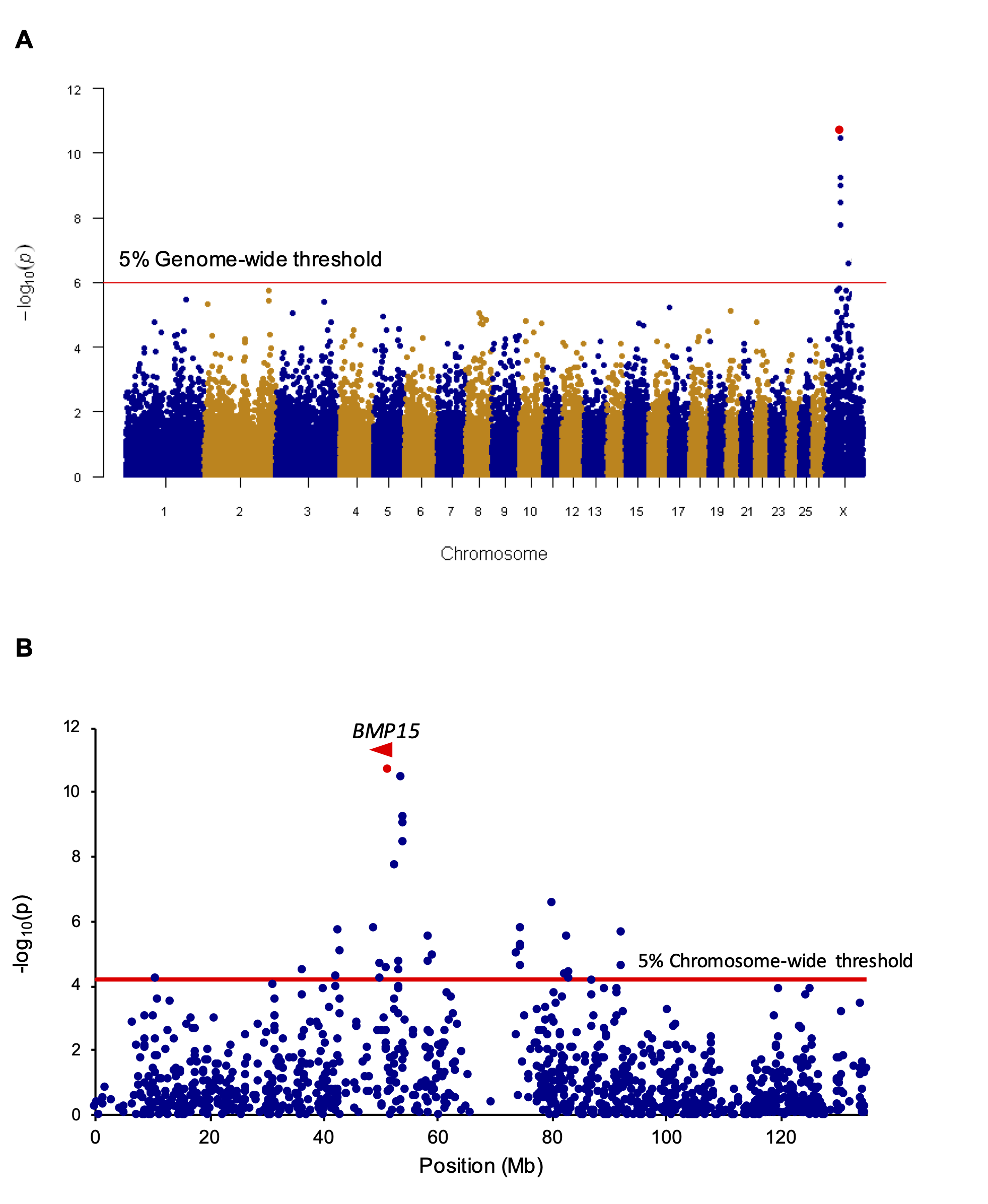

Supplement: FIGURE S1 — Genome-wide and chromosome-wide association results integrating the SNP OARX: 50977717T > A. (A) The SNP OARX: 50977717T > A genotypes were included in the SNP50 Beadchip data for genome-wide association analysis for litter size in the NV sheep population. Manhattan plot shows the combined association signals [-log10(p-value)] on the y-axis vs. SNPs position in the sheep genome on the x-axis and ordered by chromosome number (assembly OARv3.1). Red line represents the 5% genome-wide threshold. (B) OARX chromosome-wide association results. The curve shows the combined association signals [-log10(p-value)] on the y-axis vs. SNPs position on the X chromosome on the x-axis (assembly OARv3.1). Red line represents the 5% chromosome-wide threshold. In both panels, the position of the SNP OARX:50977717T > A is indicated by a red dot. In (B), the BMP15 gene location is indicated by a red arrowhead. [file Image_1.TIFF]
