# Supplementary material for: Genome-Wide Identification of a Regulatory Mutation in BMP15 Controlling Prolificacy in Sheep
Source: Front Genet. 2020 Jun 19;11:585. doi: 10.3389/fgene.2020.00585 (PMC7317000; doi:10.3389/fgene.2020.00585)
Supplement: TABLE S1 — List of variants found in the OARX: 50639087–54114793 region. Listing of 60 SNPs and 90 small INDELs with quality score > 30. [file Table_1.DOCX]

S1_Table : Polymorphisms identified in the OARX region corresponding to the shortest associated haplotype

| Position (bp) OAR X | Gene annotation | Polymorphism | Quality score | Localization | Potential impact |
| --- | --- | --- | --- | --- | --- |
| 50683780 | GSPT2-ENSOARG00000005651 | InDel | 260.8 | intergenic_region | MODIFIER |
| 50683788 | GSPT2-ENSOARG00000005651 | InDel | 260.8 | intergenic_region | MODIFIER |
| 50684673 | GSPT2-ENSOARG00000005651 | SNP | 64.6 | intergenic_region | MODIFIER |
| 50685195 | GSPT2-ENSOARG00000005651 | SNP | 180.6 | intergenic_region | MODIFIER |
| 50774266 | ENSOARG00000005651-9353 | SNP | 332.1 | intergenic_region | MODIFIER |
| 50776441 | ENSOARG00000005651-9353 | SNP | 242.1 | intergenic_region | MODIFIER |
| 50790680 | ENSOARG00000009353 | InDel | 225.0 | downstream_gene | MODIFIER |
| 50791527 | ENSOARG00000009353 | SNP | 98.3 | downstream_gene | MODIFIER |
| 50808732 | ENSOARG00000009353-CXorf67 | SNP | 108.7 | intergenic_region | MODIFIER |
| 50850358 | CXorf67-ENSOARG00000005667 | InDel | 108.7 | intergenic_region | MODIFIER |
| 50925461 | ENSOARG00000005667-BMP15 | SNP | 67.3 | intergenic_region | MODIFIER |
| 50936876 | ENSOARG00000005667-BMP15 | SNP | 217.5 | intergenic_region | MODIFIER |
| 50960665 | ENSOARG00000005667-BMP15 | SNP | 426.1 | intergenic_region | MODIFIER |
| 50967353 | BMP15 | InDel | 258.4 | downstream_gene | MODIFIER |
| 50977717 | BMP15 | SNP | 185.9 | upstream_gene | MODIFIER |
| 51169689 | ENSOARG00000005682-SHROOM4 | InDel | 600.4 | intergenic_region | MODIFIER |
| 51171209 | ENSOARG00000005682-SHROOM4 | SNP | 63.4 | intergenic_region | MODIFIER |
| 51171228 | ENSOARG00000005682-SHROOM4 | SNP | 61.38 | intergenic_region | MODIFIER |
| 51187703 | SHROOM4 | InDel | 236.4 | upstream_gene | MODIFIER |
| 51187721 | SHROOM4 | SNP | 284.5 | upstream_gene | MODIFIER |
| 51209024 | SHROOM4 | InDel | 48.8 | intron_variant | MODIFIER |
| 51218821 | SHROOM4 | InDel | 428.1 | intron_variant | MODIFIER |
| 51274978 | SHROOM4 | SNP | 179.5 | intron_variant | MODIFIER |
| 51309476 | SHROOM4 | InDel | 183.1 | intron_variant | MODIFIER |
| 51311142 | SHROOM4 | InDel | 141.8 | intron_variant | MODIFIER |
| 51373045 | SHROOM4 | InDel | 283.4 | intron_variant | MODIFIER |
| 51384668 | U6 | SNP | 64.1 | upstream_gene | MODIFIER |
| 51410216 | SHROOM4 | SNP | 193.4 | intron_variant | MODIFIER |
| 51430073 | SHROOM4 | SNP | 271.4 | intron_variant | MODIFIER |
| 51498805 | ENSOARG00000009517 | InDel | 53.0 | intron_variant &  splice_acceptor or donor | HIGH |
| 51597462 | ENSOARG00000009561 | InDel | 370.4 | downstream_gene | MODIFIER |
| 51628714 | ENSOARG00000009561-DGKK | InDel | 165.9 | intergenic_region | MODIFIER |
| 51669479 | ENSOARG00000009561-DGKK | InDel | 151.4 | intergenic_region | MODIFIER |
| 51712754 | DGKK | InDel | 435.4 | intron_variant | MODIFIER |
| 51746018 | DGKK | InDel | 228.9 | intron_variant | MODIFIER |
| 51820552 | DGKK | SNP | 665.4 | intron_variant | MODIFIER |
| 51824476 | DGKK | SNP | 44.9 | intron_variant | MODIFIER |
| 51830181 | DGKK | InDel | 78.9 | intron_variant | MODIFIER |
| 51830183 | DGKK | InDel | 82.3 | intron_variant | MODIFIER |
| 51830185 | DGKK | InDel | 82.3 | intron_variant | MODIFIER |
| 51862972 | DGKK | SNP | 267.8 | intron_variant | MODIFIER |
| 51872169 | DGKK-CCNB3 | InDel | 396.3 | intergenic_region | MODIFIER |
| 51898542 | CCNB3 | SNP | 37.4 | intron_variant | MODIFIER |
| 51964533 | AKAP4 | InDel | 87.5 | intron_variant | MODIFIER |
| 52059459 | AKAP4-CLC5 | InDel | 498.8 | intergenic_region | MODIFIER |
| 52123748 | CLC5 | SNP | 30.9 | intron_variant | MODIFIER |
| 52151774 | ENSOARG00000021616 | SNP | 46.2 | downstream_gene | MODIFIER |
| 52157764 | ENSOARG00000021616 | InDel | 166.1 | upstream_gene | MODIFIER |
| 52194284 | CLC5 | SNP | 103.9 | intron_variant | MODIFIER |
| 52194285 | CLC5 | SNP | 103.9 | intron_variant | MODIFIER |
| 52194287 | CLC5 | SNP | 103.9 | intron_variant | MODIFIER |
| 52194292 | CLC5 | InDel | 94.0 | intron_variant | MODIFIER |
| 52194293 | CLC5 | InDel | 94.0 | intron_variant | MODIFIER |
| 52195091 | CLC5 | SNP | 184.9 | intron_variant | MODIFIER |
| 52198430 | CLC5 | SNP | 126.5 | intron_variant | MODIFIER |
| 52200617 | CLC5 | InDel | 266.4 | intron_variant | MODIFIER |
| 52231408 | CLC5 | InDel | 115.8 | intron_variant | MODIFIER |
| 52232868 | CLC5 | SNP | 183.5 | intron_variant | MODIFIER |
| 52242121 | CLC5 | InDel | 96.4 | intron_variant | MODIFIER |
| 52252970 | CLC5 | InDel | 182.4 | intron_variant | MODIFIER |
| 52296947 | CLC5-USP27X | SNP | 303.4 | intergenic_region | MODIFIER |
| 52315094 | CLC5-USP27X | SNP | 135.4 | intergenic_region | MODIFIER |
| 52385758 | USP27X-PPP1R3F | InDel | 46.1 | intergenic_region | MODIFIER |
| 52388718 | USP27X-PPP1R3F | InDel | 204.2 | intergenic_region | MODIFIER |
| 52398685 | USP27X-PPP1R3F | InDel | 398.1 | intergenic_region | MODIFIER |
| 52419154 | USP27X-PPP1R3F | InDel | 246.4 | intergenic_region | MODIFIER |
| 52435375 | USP27X-PPP1R3F | InDel | 92.8 | intergenic_region | MODIFIER |
| 52435376 | USP27X-PPP1R3F | InDel | 92.8 | intergenic_region | MODIFIER |
| 52442635 | USP27X-PPP1R3F | InDel | 290.8 | intergenic_region | MODIFIER |
| 52442742 | USP27X-PPP1R3F | InDel | 99.1 | intergenic_region | MODIFIER |
| 52449513 | USP27X-PPP1R3F | InDel | 249.8 | intergenic_region | MODIFIER |
| 52450227 | USP27X-PPP1R3F | SNP | 126.8 | intergenic_region | MODIFIER |
| 52462574 | USP27X-PPP1R3F | InDel | 217.5 | intergenic_region | MODIFIER |
| 52471415 | USP27X-PPP1R3F | SNP | 595.6 | intergenic_region | MODIFIER |
| 52488251 | USP27X-PPP1R3F | InDel | 97.3 | intergenic_region | MODIFIER |
| 52488253 | USP27X-PPP1R3F | InDel | 97.3 | intergenic_region | MODIFIER |
| 52488257 | USP27X-PPP1R3F | InDel | 97.3 | intergenic_region | MODIFIER |
| 52488261 | USP27X-PPP1R3F | SNP | 107.3 | intergenic_region | MODIFIER |
| 52488265 | USP27X-PPP1R3F | SNP | 107.3 | intergenic_region | MODIFIER |
| 52503680 | USP27X-PPP1R3F | InDel | 321.1 | intergenic_region | MODIFIER |
| 52505651 | USP27X-PPP1R3F | InDel | 184.1 | intergenic_region | MODIFIER |
| 52571083 | USP27X-PPP1R3F | InDel | 265.4 | intergenic_region | MODIFIER |
| 52572019 | USP27X-PPP1R3F | InDel | 55.9 | intergenic_region | MODIFIER |
| 52572022 | USP27X-PPP1R3F | InDel | 55.9 | intergenic_region | MODIFIER |
| 52592521 | PPP1R3F | InDel | 84.8 | intron_variant | MODIFIER |
| 52634032 | CACNA1F | SNP | 95.3 | upstream_gene | MODIFIER |
| 52648352 | CACNA1F | SNP | 85.3 | intron_variant | MODIFIER |
| 52688623 | PRICKLE3 | SNP | 85.2 | synonymous_variant | LOW |
| 52694287 | PLP2 | InDel | 240.8 | upstream_gene | MODIFIER |
| 52694288 | PLP2 | InDel | 240.8 | upstream_gene | MODIFIER |
| 52699828 | MAGIX | InDel | 108.4 | intron_variant | MODIFIER |
| 52716181 | MAGIX-GPKOW | InDel | 150.8 | intergenic_region | MODIFIER |
| 52718549 | MAGIX-GPKOW | SNP | 342.8 | intergenic_region | MODIFIER |
| 52730466 | GPKOW | InDel | 115.5 | intron_variant | MODIFIER |
| 52753089 | WDR45 | InDel | 94.0 | upstream_gene | MODIFIER |
| 52758130 | PRAF2 | SNP | 148.6 | upstream_gene | MODIFIER |
| 52759563 | WDR45 | SNP | 173.9 | downstream_gene | MODIFIER |
| 52815648 | TFE3-GRIPAP1 | SNP | 232.1 | intergenic_region | MODIFIER |
| 52951821 | TIMM17B-PCSK1N | InDel | 141.4 | intergenic_region | MODIFIER |
| 53000172 | GATA1 | InDel | 367.1 | downstream_gene | MODIFIER |
| 53013209 | GLOD5 | InDel | 150.4 | downstream_gene | MODIFIER |
| 53036028 | GLOD5-SUV39H1 | InDel | 273.8 | intergenic_region | MODIFIER |
| 53037226 | GLOD5-SUV39H1 | InDel | 81.3 | intergenic_region | MODIFIER |
| 53050317 | GLOD5-SUV39H1 | SNP | 277.4 | intergenic_region | MODIFIER |
| 53066679 | GLOD5-SUV39H1 | SNP | 278.4 | intergenic_region | MODIFIER |
| 53067496 | GLOD5-SUV39H1 | InDel | 270.4 | intergenic_region | MODIFIER |
| 53073894 | GLOD5-SUV39H1 | InDel | 58.4 | intergenic_region | MODIFIER |
| 53094340 | GLOD5-SUV39H1 | InDel | 105.8 | intergenic_region | MODIFIER |
| 53111357 | GLOD5-SUV39H1 | SNP | 249.5 | intergenic_region | MODIFIER |
| 53111359 | GLOD5-SUV39H1 | InDel | 510.3 | intergenic_region | MODIFIER |
| 53111360 | GLOD5-SUV39H1 | InDel | 510.3 | intergenic_region | MODIFIER |
| 53119614 | SUV39H1 | SNP | 302.4 | downstream_gene | MODIFIER |
| 53155055 | WAS-U6 | InDel | 218.8 | intergenic_region | MODIFIER |
| 53157293 | WAS-U6 | InDel | 421.1 | intergenic_region | MODIFIER |
| 53160590 | WAS-U6 | InDel | 577.1 | intergenic_region | MODIFIER |
| 53172125 | U6 | InDel | 389.4 | upstream_gene | MODIFIER |
| 53175489 | U6-WDR13 | InDel | 89.2 | intergenic_region | MODIFIER |
| 53180238 | U6-WDR13 | InDel | 245.4 | intergenic_region | MODIFIER |
| 53183532 | U6-WDR13 | InDel | 705.4 | intergenic_region | MODIFIER |
| 53192147 | WDR13 | InDel | 232.5 | downstream_gene | MODIFIER |
| 53203613 | WDR13 | SNP | 490.8 | upstream_gene | MODIFIER |
| 53203614 | WDR13 | InDel | 481.8 | upstream_gene | MODIFIER |
| 53206594 | WDR13-RBM3 | InDel | 96.5 | intergenic_region | MODIFIER |
| 53215298 | RBM3 | InDel | 54.2 | intron_variant | MODIFIER |
| 53226133 | TBC1D25 | InDel | 173.4 | downstream_gene | MODIFIER |
| 53248717 | EBP | SNP | 297.4 | upstream_gene | MODIFIER |
| 53261035 | PORCN | SNP | 207.2 | upstream_gene | MODIFIER |
| 53280331 | PORCN-ENSOARG00000012432 | InDel | 92.8 | intergenic_region | MODIFIER |
| 53297224 | ENSOARG00000012432 | InDel | 344.3 | downstream_gene | MODIFIER |
| 53305639 | ENSOARG00000012432 | InDel | 997.3 | upstream_gene | MODIFIER |
| 53324140 | SLC38A5 | SNP | 179.6 | downstream_gene | MODIFIER |
| 53324711 | SLC38A5 | InDel | 297.4 | downstream_gene | MODIFIER |
| 53355540 | ENSOARG00000012563-ENSOARG00000012572 | SNP | 236.9 | intergenic_region | MODIFIER |
| 53372958 | ENSOARG00000012572-ZNF81 | InDel | 56.8 | intergenic_region | MODIFIER |
| 53382059 | ENSOARG00000012572-ZNF81 | SNP | 213.2 | intergenic_region | MODIFIER |
| 53438078 | ZNF81-ZNF182 | InDel | 295.4 | intergenic_region | MODIFIER |
| 53522359 | ENSOARG00000012661-ENSOARG00000005755 | SNP | 278.1 | intergenic_region | MODIFIER |
| 53559196 | ENSOARG00000012661-ENSOARG00000005755 | SNP | 32.0 | intergenic_region | MODIFIER |
| 53572746 | ENSOARG00000012661-ENSOARG00000005755 | SNP | 115.7 | intergenic_region | MODIFIER |
| 53670633 | ENSOARG00000012770-ENSOARG00000012779 | SNP | 334.6 | intergenic_region | MODIFIER |
| 53734133 | ENSOARG00000012779-UXT | InDel | 328.8 | intergenic_region | MODIFIER |
| 53734260 | ENSOARG00000012779-UXT | InDel | 218.9 | intergenic_region | MODIFIER |
| 53898797 | 5S_rRNA | SNP | 146.9 | upstream_gene | MODIFIER |
| 53917170 | 5S_rRNA-ZNF41 | InDel | 104.8 | intergenic_region | MODIFIER |
| 53939528 | 5S_rRNA-ZNF41 | SNP | 365.4 | intergenic_region | MODIFIER |
| 53992481 | ZNF41 | InDel | 91.8 | downstream_gene | MODIFIER |
| 54070439 | ZNF157 | SNP | 215.6 | upstream_gene | MODIFIER |
| 54113756 | ZNF157-ENSOARG00000005782 | InDel | 602.1 | intergenic_region | MODIFIER |
| 54113759 | ZNF157-ENSOARG00000005782 | SNP | 611.1 | intergenic_region | MODIFIER |
| 54113769 | ZNF157-ENSOARG00000005782 | InDel | 621.4 | intergenic_region | MODIFIER |
