# Supplementary material for: Genome-Wide Identification of a Regulatory Mutation in BMP15 Controlling Prolificacy in Sheep
Source: Front Genet. 2020 Jun 19;11:585. doi: 10.3389/fgene.2020.00585 (PMC7317000; doi:10.3389/fgene.2020.00585)
Supplement: TABLE S2 — List of primers used in the study. Locations of primers are based on the OARv3.1 ovine genome assembly available on ensembl.org. [file Table_2.DOCX]

S2 Table: List of primers used in the study

| Gene | Primer sequence | Position start^1^ | Application |
| --- | --- | --- | --- |
| BMP15  (NC_019484) | TGGAAGTAGGGTGGGAACAG | g.50977851 | Terra PCR *FecX*^N^ |
|  | CACAAAGGATAGGGCAAGGA | g.50977530 |  |
|  |  |  |  |
|  | GCATGGTACCTGGAAGTAGGGTGGGAACAG | g.50977851 | promotor plasmide construction |
|  | TGAAAAGCTTCACAAAGGATAGGGCAAGGA | g.50977530 |  |
|  | CCCACACAGAATCTAAACACC | g.50978310 |  |
|  | GAGGCCTTGCTACACTAGCC | g.50977109 |  |
|  |  |  |  |
|  | CCTTTTAAATACGATCTTGTTCC | g.50978310 | full length cDNA PCR amplification |
|  | CCTTTTATATACGATCTTGTTCC | g.50978310 |  |
|  | GTCACCTGCATGTGCAGGACTG | g.50977109 |  |
|  |  |  |  |
|  | GTCAAGCAGGCAGTATTG | g.50971300 | quantitative PCR |
|  | AGAATTGAGACCATAGTGTAGTA | g.50971115 |  |
|  |  |  |  |
|  | CACAAAGGATAGGGCAAGGAA | g.50977530 | sequencing |
|  | ACTTTTCTTCCCCATTTTCTCCC | g.50977044 |  |
|  | GAGGGAACAAGAGCAAAGCG | g.50971807 |  |
|  | GGCAATCATACCCTCATACTCC | g.50970959 |  |
| B4GALNT2  (NC_019468) | TGGTTCAAACTCCTACATGCAAGA | g.36938189 | Terra PCR *FecL^L^* |
|  | TATGCATGGCATGTGATAGG | g.36938314 |  |
|  |  |  |  |
|  | TATGCATGGCATGTGATAGG | g.36938314 | KASP PCR *FecL^L^* |
|  | GCAAGAAGCTGCGTGTGT | g.36938207 |  |
|  | GCAAGAAGCTGCGTGTGA | g.36938207 |  |
| GDF9  (NC_019462) | CATTTCCCCATACCAGTCTTC | g.41843543 | sequencing |
|  | CCAATCTGCTCCTACACACCT | g.41843102 |  |
|  |  |  |  |
|  | AAAACAATCCAACAGTAATGCCA | g.41842008 |  |
|  | GCTCCTCCTTACACAACACACAG | g.41840902 |  |
|  |  |  |  |
|  | TAGTCAGCTGAAGTGGGACA | g.41841090 | quantitative PCR |
|  | AGCCATCAGGCTCGATGGCC | g.41841294 |  |
| GAPDH  (NM_001190390) | CGACTTCAACAGCGACACTC | c.885 | quantitative PCR |
|  | CAATGAATTCGGCTACAGCA | c.978 |  |
| SDHA  (XM_027980212) | GAATGGTCTGGAACACTG | c.1818 |  |
|  | AGTAATCGTACTCGTCAAC | c.1955 |  |

^1^Genomic start positions of primers (in base pair) localized on the OARv3.1 ovine genome assembly
